# Development of a short form of the Spanish schedule of attitudes toward hastened death in a palliative care population

**DOI:** 10.1007/s11136-016-1409-0

**Published:** 2016-09-26

**Authors:** Cristina Monforte-Royo, Luis González-de Paz, Joaquín Tomás-Sábado, Barry Rosenfeld, Julia Strupp, Raymond Voltz, Albert Balaguer

**Affiliations:** 1 Department of Nursing, School of Medicine and Health Sciences, Universitat Internacional de Catalunya, Sant Cugat del Vallés, Barcelona Spain; 2 Public Health Unit, School of Medicine and Health Sciences, Universitat Internacional de Catalunya, Sant Cugat del Vallés, Barcelona Spain; 3 Centre d’Atenció Primària Les Corts. Transverse Group for Research in Primary Care, IDIBAPS, Barcelona, Spain; 4 Escola Universitària d’Infermeria Gimbernat, Autonomous University of Barcelona, Barcelona, Spain; 5 Department of Psychology, Fordham University, Bronx, NY USA; 6 Department of Palliative Medicine, University Hospital of Cologne, Cologne, Germany; 7 School of Medicine and Health Sciences, Universitat Internacional de Catalunya, Josep Trueta s/n, 08195 Sant Cugat del Vallès, Barcelona Spain

**Keywords:** Wish to hasten death, Desire to hasten death, Desire to die, Palliative care, End-of-life care, End-of-life decisions, Measurement, Instrument, Item response theory

## Abstract

**Purpose:**

The schedule of attitudes toward hastened death (SAHD) is widely used to assess the wish to hasten death (WTHD) among patients with life-threatening conditions. A short form of the SAHD would increase its clinical applicability in this population.

**Method:**

Rasch analysis of data from 101 Spanish palliative inpatients. Item reduction involved selecting items with a high discrimination index (point-biserials ≥0.70), removing items with inadequate fit statistics, and assessing unidimensionality and local dependency. We examined the test probability function to establish an empirical risk score for suffering a WTHD and tested convergence between the original and the reduced set of items.

**Results:**

A set of five items met all quality criteria. In this sample, 20.8 % of participants had a higher risk of a WTHD (*p* > 50 %) at a score of 3. Correlation analysis confirmed convergent validity between the original and reduced forms. Concurrent validity was confirmed by the similar correlations shown by both versions of the SAHD (5 and 20 items) with other measures.

**Conclusion:**

This 5-item Spanish form of the SAHD could be a suitable alternative to the full instrument. The cut-off score derived from the Rasch analysis may be able to detect patients at risk of a WTHD.

## Introduction

The wish to hasten death (WTHD) is a reaction to a suffering which can occur in patients with life-threatening conditions [1, 2]. In the last two decades, several instruments have been designed to measure the WTHD and to identify factors related with it [3–6]. The most widely used instrument is the schedule of attitudes toward hastened death (SAHD). Developed by Rosenfeld et al. [7] the SAHD is a self-report questionnaire with 20 items that respondents must rate as true or false. The psychometric properties of the SAHD were originally studied in North American samples involving patients with AIDS [7] and palliative care (PC) cancer patients [4]. The SAHD has been successfully adapted into several languages [8–11], including Spanish [12].

Although the SAHD has become a well-established instrument for research purposes [7] some of its characteristics make it less suitable for use with patients who are physically or emotionally fragile. For example, the task of responding to 20 items may be exhausting for some patients [10, 12, 13]. Furthermore, while a clinically defined cut-off score for “high” desire to die has been proposed previously [13], a score for predicting the risk of a WTHD has yet to be established.

This study therefore had two aims: to develop a short form of the Spanish SAHD and to define, for this short form, a risk score for the WTHD in PC patients, using a stochastic model based on scores from the SAHD.

## Materials and methods

We conducted an item reduction study of the Spanish version (SV) of the SAHD (SAHD-20 SV). Data came from a previous cross-sectional study [12] in which a convenience sample of 101 patients (62 male; *M*_age_ 61.7 ± 11 years, range 33–84) from a PC oncology unit in Barcelona (Spain) completed the SAHD-20 SV. The sample is described in detail elsewhere [12].

### Data analysis

We used a Rasch model to reduce the number of SAHD-20 SV items and to analyse whether the new set of items would retain the information needed to detect a risk for the WTHD in PC patients. The procedure involved three steps, in each of which a Rasch analysis was carried out iteratively: (1) after fitting data to the dichotomous Rasch model we selected items with high discrimination values (point-biserials ≥0.70). Point-biserials are Pearson’s product-moment correlations between each item location and the mean Rasch item estimation, excluding the item being analysed [14]. Higher values would correspond to a stronger WTHD construct. (2) Items with fit statistics (Infit and Outfit) outside the range 0.7–1.4 were examined and eventually removed. These goodness-of-fit statistics indicate how accurately or predictably the data fit the model [15]. (3) The person separation index—analogous to Cronbach’s alpha (reliability)—and the unidimensionality assumption of the final set of items were examined. A paired *t* test for the difference between two sets of items identified by a residual-based principal components analysis (PCAR) was made at the person level; the percentage of these tests outside the range of the 95 % confidence interval was not expected to exceed 5 %. If the Rasch quality criteria were satisfied, we examined the test probability function of the reduced set of items to find a score that would indicate patients with a ≥50 % risk of having a WTHD according to the new short version. In addition, the strength of the relationship between the reduced SAHD and the SAHD-20 SV was examined using Pearson’s correlations. In order to assess correlations with factors associated with the WTHD, we also examined the correlations of both the reduced (SAHD-5 SV) and 20-item versions of the SAHD with other instruments (all Spanish versions): Karnofsky Performance Status (KPS) [16], where higher scores [0–100] indicate a healthier status; the Barthel Index (BI) [17], to assess the degree of independence in activities of daily living (range 0–100, with higher scores indicating greater independence); the Eastern Cooperative Oncology Group Performance Status (ECOG-PS) [18] (range 0–4, with higher scores indicating poorer function); the Palliative care Outcome Scale (POS) [19], to assess patients’ palliative needs (range 0–44, with higher scores indicating greater needs); and the Hospital Anxiety and Depression Scale (HADS) [20], to assess psychological distress (range 0–21 for both anxiety (HADS-A) and depression (HADS-D), with higher scores indicating levels of anxiety or depression requiring specialist assessment).

## Results

The first step of the reduction process showed that only seven items had point-biserial correlations >0.70. Table 1 shows the correlations between item measures and the mean Rasch item measures. In the second step (goodness-of-fit analysis), two of these items (20 and 16) were removed as their Infit and Outfit values were outside the established range; inspection of these two items suggested that the most likely cause of the misfit was the use of a double-barrelled statement (item 20) and a threatening wording that might drive participants to respond in a contrary way (item 16). The five remaining items had adequate goodness-of-fit values. Table 2 shows the item location values of the final five items and the goodness-of-fit statistics, as well as the two items removed in each of the analyses. Reliability of the final five-item scale was adequate (item separation index = 0.7). The percentage of individual *t* tests outside the range ±1.96 between items with positive and negative loadings from the PCAR was optimal (<5 %), thus confirming the hypothesis that the five items of the reduced scale (SAHD-5 SV) could be aligned on the same latent trait continuum. Participants’ mean score on the SAHD-5 SV was −1.72 logits (SE = 0.21). Figure 1 shows a plot of the test characteristic curve, or the sum of the item information functions, representing the Rasch model prediction for each measure relative to the WTHD when using the new SAHD-5 SV. The dashed lines indicate that the risk (≥50 %) of suffering a WTHD corresponds to patients who scored >2 on the SAHD-5 SV. Using this score and the SAHD-5 SV, 21 (20.8 %) participants had a risk of having a WTHD.

**Table 1:** Correlations between item measures of the SAHD and the mean Rasch item measure estimation

Item	Point-biserial adjusted correlation
**4.**	**0.80**
**14.**	**0.79**
**16.**	**0.78**
**13.**	**0.77**
**10.**	**0.76**
**20.**	**0.76**
**12.**	**0.73**
6.	0.68
11.	0.62
8.	0.58
7.	0.57
19.	0.57
3.	0.56
18.	0.56
9.	0.55
5.	0.50
2.	0.43
15.	0.41
17.	0.27
1.	0.26

Bold Values indicate, the items with point-biserial correlations >0.70

**Table 2:** Item location values of the final five items and the corresponding goodness-of-fit statistics (items are True/False in the schedule of attitudes toward hastened death^a^), plus the two items (20 and 16) removed in each of the analyses

Reduced SAHD Version	Item	Location (error)	Infit	Outfit
Final	**4.** I am seriously considering asking my doctor for help in ending my life. (True)	1.45 (0.53)	0.83	1.19
	**10.** I hope my disease will progress rapidly because I would prefer to die rather than continue living with this illness. (True)	0.24 (0.47)	0.79	0.87
	**12.** I enjoy my present life, even with my illness, and would not consider ending it. (False)	0.24 (0.47)	1.24	1.23
	**13.** Because my illness cannot be cured, I would prefer to die sooner, rather than later. (True)	−0.63 (0.47)	0.96	1.23
	**14.** Dying seems like the best way to relieve the emotional suffering my illness causes. (True)	−1.29 (0.48)	1.02	0.85
*2*	***20.*** *I am able to cope with the symptoms of my illness and have no thoughts of ending my life.*	−1.04 (0.44)	1.5	1.44
*1*	***16.*** *Because of my illness, the idea of dying seems comforting.*	−2.86 (0.66)	1.00	1.95

^a^In brackets, true or false indicates the response that would indicate more desire for death

Correlation between the Rasch measures of the SAHD-5 SV and scores on the SAHD-20 SV was strong (*r* = 0.944, *p* < 0.01), and both versions of the SAHD (5 and 20 items) produced similar correlations with the other measures (Table 3).

**Table 3:** Pearson coefficients for SAHD-5 SV and SAHD-20 SV [12] in relation to the measures of performance status: Barthel Index [17], Karnofsky Performance Status [16], and Eastern Cooperative Oncology Group (ECOG) [18], Palliative Outcome Scale (POS) [19], and Hospital Anxiety and Depression Scale (HADS) [20]

	SAHD 20 item	Barthel index	ECOG	Karnofsky	POS	HADS total	HADS anxiety	HADS depression
SAHD 5 items	0.944**	−0.398**	0.311**	−0.373**	0.499**	0.512**	0.259**	0.565**
SAHD 20 items	1	−0.400**	0.319**	−0.403**	0.542**	0.514**	0.269**	0.559**

** *p* < 0.01

## Discussion

The results suggest that the proposed 5-item scale could be a valid and reliable instrument for assessing and detecting the risk of a WTHD. If so, the item reduction would confer an important advantage when assessing the WTHD in the most fragile patients.

The reduction process (similar to that used by Rosenfeld et al. [21] in the recently published English short form) drew attention to the possible influence of double-barrelled wording on patients’ understanding of some SAHD-20 SV items, the scores on which might therefore reflect two issues rather than just one as intended. This aspect, not highlighted in previous research [12], is likely due to the fact that Rasch analysis is the most restrictive model within IRT; other IRT models–such as 2PL or 3PL—might be less restrictive and would fit these items. It should also be noted that the psychometric properties of all other adaptations of the SAHD were originally examined using methods derived from classical test theory, which does not stress the study of individual items, unlike the approach taken here.

Clearly, the item reduction entails a certain loss of information. However, correlations with other instruments used to assess patients’ physical and psychological status were comparable for the Spanish SAHD-5 and SAHD-20 (see Table 3). Moreover, the SAHD-5 SV contains a feasible number of items for obtaining the information needed to assess the risk of a WTHD in clinical practice.

Assessment of the WTHD by means of the SAHD-5 SV identified 20.8 % of patients as being at risk of a WTHD (score >2), whereas the SAHD-20 SV [12] detected 16.8 % of patients with a score considered “high” WTHD in the literature. The short form thus appears to show increased sensitivity for detecting the WTHD. Because Rasch analysis is a probabilistic model it allows us to estimate the “amount” of WTHD among participants. The usual criterion in this context is that the probability or risk of presenting a given construct is high if the respondent scores ≥50 % on the measure used to assess that construct. This approach contrasts with previous research in which labels of ‘*high’*, ‘*moderate’* or ‘*low’* WTHD were established clinically, without using a measurement model or subsequent verification. Our results therefore suggest that the SAHD-5 SV might be useful for detecting the risk of a WTHD in PC patients. Further research is, however, necessary to evaluate the effectiveness of the instrument in similar patient populations, to clarify criteria for labelling a patient as experiencing a WTHD or not, and, thereafter, to assess the sensitivity and specificity of the new instrument using suitable methods (e.g. ROC curves).

This study has several limitations. One derives from the nature and purpose of the SAHD-20 SV, which meant that data were obtained from a relatively small convenience sample consisting solely of cancer patients. The lack of a gold standard for assessing the specificity and sensitivity of the proposed short form is a further limitation. However, data goodness of fit to the Rasch model was optimal. Furthermore the use of the Rasch model allowed us to convert discrete units into intervals, and thus the SAHD-5 SV could be used to study the sensitivity and specificity of other instruments. Another limitation concerns the weak to moderate correlation obtained for some of the scales that assess physical status or daily function. Other scales that examine psychological aspects such as meaning in life or hopelessness would likely have been more appropriate here.

By analysing the SAHD-20 SV at the item level, we were able not only to produce a short form in Spanish but also to identify some potential shortcomings in the original SAHD. We therefore believe that our approach and results will be of interest to researchers from other countries who are seeking to adapt this instrument or to interpret their own results, since the functioning of the SAHD is likely to be influenced by cultural factors.

## Conclusion

The SAHD-5 SV may constitute a valid and reliable instrument for assessing and detecting the risk of the WTHD. As such, it could contribute to the development of more effective care plans for PC patients and be used as an outcome measure of intervention studies.
